# Prevalence of selected clinical problems in older adults with autism and intellectual disability

**DOI:** 10.1186/1866-1955-5-27

**Published:** 2013-09-25

**Authors:** Dmitry Kats, Leslie Payne, Morgan Parlier, Joseph Piven

**Affiliations:** 1 Carolina Institute for Developmental Disabilities, University of North Carolina at Chapel Hill, Campus Box 7255, Chapel Hill, NC 27599-7255, USA; 2 Department of Psychiatry, University of North Carolina at Chapel Hill, Chapel Hill, NC, USA; 3 Department of Epidemiology, Gillings School of Global Public Health, University of North Carolina at Chapel Hill, Chapel Hill, NC, USA

**Keywords:** Autism, ASD, Older adults, Clinical problems, Behavior problems, Intellectual disabilities

## Abstract

**Background:**

Originally described as a disorder of childhood, evidence now demonstrates the lifelong nature of autism spectrum disorder (ASD). Despite the increase of the population over age 65, older adults with ASD remain a scarcely explored subpopulation. This study set out to investigate the prevalence of clinically relevant behaviors and medical problems in a sample of US adults aged 30 to 59 with ASD and intellectual disability (ID), in comparison to those with ID only.

**Methods:**

A cross-sectional study, with both an exploratory and replication analysis, was conducted using National Core Indicators (NCI) multi-state surveys from 2009 to 2010 and 2010 to 2011. There were 4,989 and 4,261 adults aged 30–59 with ID examined from the 2009 to 2010 and 2010 to 2011 samples, respectively. The two consecutive annual samples consisted of 438 (9%) and 298 (7%) individuals with ASD and ID. Variables were chosen from the NCI data as outcomes, including medication use for behavior problems, severe or aggressive behavior problems and selected medical conditions.

**Results:**

No age-associated disparities were observed between adults with ASD and ID versus adults with ID only in either sample. For the 2009 to 2010 sample, the prevalence of support needed to manage self-injurious, disruptive and destructive behavior in subjects with ASD and ID ranged from 40 to 60%. Similarly, the prevalence estimates of self-injurious, disruptive and destructive behavior were each almost double in adults with ASD and ID relative to those with ID only. These results were replicated in the 2010 to 2011 sample.

**Conclusions:**

The findings of this study highlight the urgent need for research on the nature and treatment of severe behavior problems in the rapidly increasing population of older adults with ASD. They also suggest the importance of developing policies that expand our capacity to care for these individuals.

## Background

Autism spectrum disorder (ASD) is a behavioral disorder defined by the presence of social deficits and repetitive behaviors with onset in early childhood [1]. The prevalence of ASD in children in the US, historically thought to be rare, is now estimated at approximately one in eighty-eight [2]. Initially described as a disorder of infancy [3], more recent papers have begun to focus on young adults. However, although reports now document that individuals with ASD live into older age [4-6], almost no systematic studies targeting individuals with ASD at older ages (that is, over age 50 years) have been reported.

US Census Bureau estimates in 2006 projected a doubling of the US population over 65 years of age by the year 2030. Assuming the life expectancy of individuals with ASD is similar to that of the general population, based on current prevalence rates of ASD in school-age children, this population expansion would result in approximately 700,000 individuals with ASD over 65 years of age in less than 20 years. With the aging of the ASD population in western countries, increasing rate of diagnosis of ASD and burgeoning use of services by persons with ASD, the need to learn more about aging and autism is a high priority. Estimated per capita lifetime direct and indirect costs of ASD in the US are over $3 million and do not include expenses related to individuals with ASD living into their 60s and older [7]. The need to look beyond younger affected populations in efforts to mitigate the burdens of ASD is highlighted by recent analyses noting that the largest contributor to the estimated per capita lifetime direct cost of ASD is care during adulthood [7]. A better understanding of the behavioral progression, associated medical problems and care needs of older adults with autism is critical for addressing this approaching major public health problem. Additionally, gaining knowledge of lifetime trajectories of ASD is likely to provide important clues to teasing apart the clinical and etiologic heterogeneity of this condition. This knowledge will also provide needed information on the trajectories of care from family to institutional care patterns and needs as ASD patients move through their lifecycle.

This study takes advantage of a large, nationwide dataset that includes selected behavioral and medical outcomes in adults of age 18 years and older, with clinical diagnosis of both ASD and intellectual disability (ID). The opportunity to examine older adults with both ASD and ID afforded the chance to study those older ASD individuals likely to have the greatest degree of functional impairment. Estimates of the prevalence of ID in individuals with ASD range from 38% in children with ASD [2] to 70% in all those with ASD [8]. Contrasting those individuals with both ASD and ID to those with ID but not ASD allowed us to examine behavior-related, neurological and sensory outcomes that were specific to the aging ASD population.

## Methods

### Data source

The National Core Indicators (NCI) effort comprises collaboration between the National Association of State Directors of Developmental Disability Services and the Human Services Research Institute (HSRI) [9] to produce a dataset that assesses performance in the delivery of state services to adults with a developmental disability. The core indicators include approximately 100 demographic and outcome metrics, such as aspects of physical and mental health, receipt of selected services and access to care, thought to be important for measuring performance of public agencies working in the area of developmental disabilities. For the NCI effort, data are collected annually on a random sample of approximately 400 adults ≥18 years of age (in each of the participating states/geographic areas), who have an intellectual and/or developmental disability and have received at least one state service in addition to case management during the fiscal year. NCI workers acquire the data from sources of information including medical and state records, as well as interviews with providers, family members and clients for the Background Information Section (specified below). Data are then compiled into a single dataset maintained by the HSRI.

Data from the 2009 to 2010 and 2010 to 2011 multi-state merged NCI surveys were obtained from the HSRI after a detailed application process. To maximize confidentiality, within the merged dataset for each year, there is no variable to indicate the state of origin of the subjects. In addition, subjects were de-identified and were not assigned a unique identification number, thus making it impossible to track whether subjects appeared more than once within the two years of data obtained. Conversations with HSRI officials suggested that multiple individual entries were less likely in more populated states than in states with fewer subjects. Lacking certainty about the possibility of minimal duplication of individuals, it was decided that the data should not be merged across the multi-year sampling frame but rather investigated one year at a time. A cross-sectional method was therefore employed using the 2009 to 2010 data to conduct an exploratory analysis and subsequent 2010 to 2011 data to provide a replication sample.

Although 24 states currently participate in the NCI effort, data for the exploratory study were from 11,599 subjects from the 18 regions that administered the Adult Consumer Survey in 2009 to 2010: Alabama, Arkansas, Washington, DC, Georgia, Illinois, Kentucky, Louisiana, Maine, Missouri, North Carolina, New Jersey, New York, Ohio, Oklahoma, Pennsylvania, Texas, Wyoming and Orange County, California. Data for the replication study were from 8,796 individuals from the 15 geographic areas that administered the Adult Consumer Survey in 2010 to 2011: Alabama, Arkansas, Florida, Georgia, Illinois, Kentucky, Louisiana, Maine, Missouri, North Carolina, New Hampshire, New York, Ohio, Oklahoma and Pennsylvania. State sampling strategies for the 2009 to 2010 and 2010 to 2011 samples appear in Appendix B of the 2009 to 2010 and 2010 to 2011 *Consumer Outcomes Final Report*[10,11], respectively.

### National core indicators

The Background Information Section of the NCI Consumer Survey served as the source of all variables used in this study. This section consisted of measures such as the service user's demographics, functioning, clinical diagnoses, general health, problem behaviors, living arrangements and services. These variables, including diagnosis of ASD, were almost always obtained from agency records or information systems, and only occasionally by case managers or surveyors who interviewed the service user and/or an informant. Each state considered a diagnosis of ASD as any of the conditions listed as pervasive developmental disorders in the *Diagnostic and Statistical Manual of Mental Disorders* (4th ed.; *DSMI-IV*; American Psychiatric Association, 1994) [12] including medical record entries (for example, autism, Asperger’s disorder or pervasive developmental disorder).

The emphasis of the current study was on medical and behavioral outcomes. Within this domain, variables were selected for inclusion in this study based on decisions made by the research team on the face validity of the information obtained. Variables that may be of potential interest, ‘medication for mood disorders?’ , ‘medication for anxiety?’ and ‘medication for psychotic disorders?’ , were not deemed of sufficient validity for inclusion because specific definitions (for example, the list of anxiety medications) were not provided for such determinations. After detailed examination, the following variables, as they appear in the NCI data, were chosen as outcomes in this study: (1) ‘medication for behavior problems?’ , (2) ‘limited or no vision-legally blind’ , (3) ‘hearing loss-severe or profound’ , (4) ‘physical disability’ , (5) ‘seizure disorder or neurological problem’ , (6) ‘Alzheimer’s disease or other dementia’ , (7) ‘does this person need support to manage self-injury behavior?’ , (8) ‘does this person need support to manage disruptive behavior?’ and (9) ‘does this person need support to manage destructive behavior?’ For outcomes (2) to (6) a clinical diagnosis of the condition was inferred to have been retrieved from the subject’s medical history or record.

Responses to these variables appeared as ‘yes’ , ‘no’ , ‘don’t know’ or ‘unknown’ , except for those requiring an assessment of the level of support needed to manage self-injurious, disruptive or destructive behavior. These latter responses were characterized as: ‘extensive support needed’ , ‘some support needed’ , ‘no support needed’ or ‘don’t know’. Due to a small proportion (<10%) of subjects in this sample for whom ‘extensive support needed’ was indicated, the responses ‘extensive support needed’ and ‘some support needed’ were combined into one category as the ‘yes’ response. Accordingly, ‘no support needed’ served as the ‘no’ response. For all variables included in this study, any entries of ‘don’t know’ or ‘unknown’ were treated as missing.

### Sample

The multi-state NCI sample from each year was largely limited to individuals diagnosed with mental retardation, referred to here as ID. In the 2009 to 2010 dataset, for instance, of the 10,935 subjects with relevant diagnostic information available, 10,627 (97.2%) had a diagnosis of ID. The present study was limited to those individuals with ID, with or without a concurrent ASD diagnosis. Diagnoses of both ID and ASD were reported in subjects’ case records and presumably established by a qualified clinician in the community beforehand. Individuals with ID and ASD (referred to in this study as the ASD group) served as the exposed group; those with ID without ASD (referred to as the ID-only group) served as the comparison group. The dataset also included levels of ID (mild, moderate, severe, profound or unspecified). Individuals with profound or unspecified levels of ID were excluded from this study. The exclusion of individuals with profound ID was based on the complexities introduced in the diagnosis of ASD in individuals with such a severe degree of intellectual impairment, raising questions about the validity of ASD diagnosis in these individuals. This study was exempt from Institutional Review Board approval.

The primary focus of this study was on medical/behavioral problems in older adults with ASD. Given the limited number of subjects in the database with ASD who were at least 60 years of age, the target population of the study was limited to adults 50 to 59 years of age. To provide insight into potential age effects that might differ in this age group relative to younger adults with ASD and ID, additional age groups of individuals 30 to 39 and 40 to 49 years of age were included.

### Statistical analyses

To model the association between autism/ASD and each of the selected outcomes, a separate Poisson regression model with a robust error variance/sandwich term [13] was developed. The use of Poisson regression with a sandwich error term, which produces prevalence ratios (PRs), is a widely preferred statistical method to estimate measures of effect when using cross-sectional data for outcomes of higher prevalence [14-17]. This form of Poisson regression provides similar measure-of-effect estimates to other modeling techniques for rare outcomes [14] and so was also used for the lower-prevalence outcomes in this study. Since age group, gender and level of ID may potentially confound the relationship between ASD and all outcomes selected for this study [2,5,18], these covariates were controlled for within the modified Poisson regression model for each outcome of interest. Adjusting for these potential confounders, prevalence ratios between the ASD and ID-only groups on each selected outcome were deemed significant at a two-tailed value of *α* = 0.05. Age group, gender and level of ID interaction terms were also tested for significance in each model at a conservative two-tailed value of *α* = 0.10, given the relatively smaller sample sizes in several of the subgroups stratified by age and level of ID.

Poisson regression models with robust error-variance were additionally utilized to examine whether these covariates were associated with each of the selected outcomes differently across the ASD and ID-only diagnostic groups. The data for this paper were generated using SAS software, Version 9.3 of the SAS system for Windows. Copyright © 2011 SAS Institute Inc. SAS; all other SAS Institute Inc. product or service names are registered trademarks or trademarks of SAS Institute Inc., Cary, NC, USA).

## Results

### Exploratory sample (2009 to 2010)

#### Participants

The 2009 to 2010 cross-sectional sample of 4,989 adults aged 30 to 59 years with mild, moderate or severe ID consisted of 438 (9%) individuals in the ASD group and 4,551 (91%) adults in the ID-only group (Table 1). In total, there were 1,516 (30%) individuals of 30 to 39 years of age, 1,808 (36%) of 40 to 49 years of age and 1,665 (33%) of 50 to 59 years of age. Mean age was 42 (SD 8) years and 45 (SD 8) years for those in the ASD group and ID-only group, respectively. Approximately 75% of the ASD group and just over half of the ID-only group were male. Severity of ID varied from 27% with mild and 37% with moderate ID to 36% with severe ID in the ASD group; and, 47% with mild and 33% with moderate ID to 19% with severe ID in the ID-only group. The majority of adults in the ASD group (75%) and ID-only group (72%) were classified as white, non-Hispanic.

**Table 1:** Demographic variables among the ASD group and the ID-only group in the 2009 to 2010 NCI sample of adults aged 30-59 years

**Demographic variables**	**ASD group**^**a**^	**ID-only group**^**b**^
	**(n = 438), number (%)**	**(n = 4,551), number (%)**
**Age**		
30 to 39 years	190 (43)	1,326 (29)
40 to 49 years	154 (35)	1,654 (36)
50 to 59 years	94 (21)	1,571 (35)
Missing data	0	0
**Gender**		
Female	117 (27)	2,008 (44)
Male	319 (73)	2,532 (56)
Missing data	2	11
**Race/ethnicity**		
White, non-hispanic	326 (75)	3,280 (72)
Black, non-hispanic	84 (19)	914 (20)
Hispanic	14 (3)	228 (5)
Asian	4 (1)	48 (1)
American Indian	2 (0)	44 (1)
Other	3 (1)	15 (0)
Missing data	5	19
**Marital status**		
Single, never married	431 (100)	4,257 (95)
Single, married in the past	0 (0)	142 (3)
Married	1 (0)	111 (2)
Missing data	6	41
**Level of ID**		
Mild	107 (27)	2,055 (47)
Moderate	147 (37)	1,441 (33)
Severe	146 (37)	840 (19)
Missing data	38	215
**Primary language**		
English	433 (99)	4,440 (98)
Other	5 (1)	95 (2)
Missing data	0	16
**Primary means of expression**		
Spoken	280 (64)	3,933 (87)
Gesture/body language	137 (31)	477 (11)
Sign language/finger spelling	13 (3)	61 (1)
Communication aid/device	3 (1)	21 (0)
Other	3 (1)	33 (1)
Missing data	2	26

^a^ASD group: diagnosis of intellectual disability (ID) and autism spectrum disorder (ASD); ^b^ID-only group: diagnosis of ID but no diagnosis of ASD. NCI, National Core Indicators.

### Outcomes

Nearly 50% of subjects in the ASD group and 25% of those in the ID-only group were prescribed medications for behavioral problems (Table 2). There were substantially higher percentages of subjects in the ASD group needing support for behavioral problems compared to the ID-only group across all three types of problem behaviors examined, with proportions in individuals with ASD and ID that were generally two to three times those reported in individuals with ID only. Four percent of the ASD group and 6% of the ID-only group were reported as limited or no vision-legally blind, and a limited number of individuals in both groups were also reported as having hearing loss-severe or profound. Slightly less than 5% of individuals in the ASD group and about 10% of members of the ID-only group were reported to have a physical disability. The presence of a seizure disorder or neurological problem was reported in approximately 20% of the ASD group compared to 25% of the ID-only group. Finally, Alzheimer’s disease or other dementia was noted in 2% of the ASD group and 1% of the ID-only group.

**Table 2:** Outcomes among the ASD group and ID-only group in the 2009 to 2010 NCI sample of adults aged 30-59 years

**Outcomes**	**ASD group**^**a**^	**ID-only group**^**b**^
	**(n = 438), number (%)**	**(n = 4,551), number (%)**
**Medication for behavior problems?**		
Yes	174 (42)	1,075 (25)
No	240 (58)	3,179 (75)
Missing data	24	297
**Limited or no vision-legally blind**		
Yes	19 (4)	259 (6)
No	419 (96)	4,292 (94)
Missing data	0	0
**Severe or profound hearing loss**		
Yes	16 (4)	217 (5)
No	422 (96)	4,334 (95)
Missing data	0	0
**Physical disability**		
Yes	19 (4)	448 (10)
No	419 (96)	4,103 (90)
Missing data	0	0
**Seizure disorder or neurological problem**		
Yes	91 (21)	1,227 (27)
No	347 (79)	3,324 (73)
Missing data	0	0
**Alzheimer's disease or other dementia**		
Yes	9 (2)	56 (1)
No	429 (98)	4,495 (99)
Missing data	0	0
**Does this person need support to manage self-injury behavior?**		
Extensive	46 (11)	174 (4)
Some	120 (28)	605 (14)
None	262 (61)	3,647 (82)
Missing data	10	125
**Does this person need support to manage disruptive behavior?**		
Extensive	52 (12)	372 (8)
Some	164 (38)	1,374 (31)
None	214 (50)	2,696 (61)
Missing data	8	109
**Does this person need support to manage destructive behavior**?		
Extensive	44 (10)	202 (5)
Some	114 (27)	821 (19)
None	270 (63)	3,404 (77)
Missing data	10	124

^a^ASD group: diagnosis of intellectual disability (ID) and autism spectrum disorder (ASD); ^b^ID-only group: diagnosis of ID but no diagnosis of ASD. NCI, National Core Indicators.

No interaction terms related to age group, gender or level of ID were significant additions to the model for any outcome (*P* >0.10), indicating that the effect of a diagnosis of ASD on the prevalence estimates of the outcomes of interest was not specific to any age group, gender or level of ID. Therefore, a separate Poisson regression model with a sandwich error term was developed for the analysis of each selected outcome across the entire sample of individuals aged 30 to 59 years. Prevalence ratios, adjusted for age group, gender and level of ID, with 95% CIs, are reported in Table 3.

**Table 3:** Prevalence ratios (ASD group versus ID-only group) for 2009 to 2010 NCI sample of adults aged 30-59 years

**Outcomes**	**Prevalence ratio (95% CI)**
Medication for behavior problems	1.11 (1.07, 1.15)
Limited or no vision-legally blind	0.98 (0.96, 1.00)
Severe or profound hearing loss	0.99 (0.97, 1.01)
Physical disability	0.95 (0.93, 0.97)
Seizure disorder or neurological problem	0.93 (0.90, 0.96)
Alzheimer's disease or other dementia	1.01 (1.00, 1.03)
Does this person need support to manage self-injury behavior?	1.87 (1.62, 2.17)
Does this person need support to manage disruptive behavior?	1.25 (1.12, 1.39)
Does this person need support to manage destructive behavior?	1.38 (1.19, 1.59)

Prevalence ratios were adjusted for age group (30 to 39, 40 to 49, 50 to 59 years), gender and level of intellectual disability (ID). ASD group: diagnosis of ID and autism spectrum disorder (ASD); ID-only group: diagnosis of ID but no diagnosis of ASD. NCI, National Core Indicators.

Prevalence estimates for the categories, medication for behavior problems (PR 1.11, 95% CI 1.07, 1.15); does this person need support to manage self-injury behavior? (PR 1.87, 95% CI 1.62, 2.17); does this person need support to manage disruptive behavior? (PR 1.25, 95% CI 1.12, 1.39); and does this person need support to manage destructive behavior? (PR 1.38, 95% CI 1.19, 1.59), were all significantly higher in the ASD group relative to the ID-only group. Notably, the ASD group had almost twice the prevalence for the category, does this person need support to manage self-injury behavior?, compared to the ID-only group. The prevalence of physical disability (PR 0.95, 95% CI 0.93, 0.97) and seizure disorder or neurological problem (PR 0.93, 95% CI 0.90, 0.96) was significantly lower in the ASD group relative to the ID-only group. There were no significant differences between the two groups for the categories, limited or no vision-legally blind (PR 0.98, 95% CI 0.96, 1.00); hearing loss-severe or profound (PR 0.99, 95% CI 0.97, 1.01); or Alzheimer’s disease or other dementia (PR 1.01, 95% CI 1.00, 1.03).

More severe level of ID was significantly associated with medication for behavior problems?; does this person need support to manage self-injury behavior?; and seizure disorder or neurological problem, in both the ASD group and ID-only group. A significant relationship between age group and hearing loss-severe or profound was observed within both groups as well, with slightly elevated prevalence estimates of hearing problems reported with increasing age. Furthermore, increasing severity of ID was significantly associated with the categories, does this person need support to manage disruptive behavior?; does this person need support to manage destructive behavior?; limited or no vision-legally blind; and physical disability, within the ID-only group but not the ASD group. None of the covariates in this study (age group, gender or level of ID) were associated with Alzheimer’s disease or other dementia in either the ASD or ID-only group. Gender was not associated with any outcomes in either group. All other possible associations between any of the outcomes and covariates, whether bivariate or multivariate, were non-significant (*P* >0.05).

### Replication sample (2010 to 2011)

#### Participants

To reflect whether the results from the 2009 to 2010 sample could be replicated, the 2010 to 2011 cross-sectional sample of 4,261 adults aged 30 to 59 years, with mild, moderate or severe ID was examined (Table 4). This sample was made up of 298 (7%) individuals in the ASD group and 3,963 (93%) adults in the ID-only group, consisting of 1,439 (34%) subjects 30 to 39 years of age, 1,573 (37%) 40 to 49 years of age and 1,249 (29%) 50 to 59 years of age. Members of the ASD group were of mean age 41 (SD 8) years, and the mean age of those in the ID-only group was 44 (SD 8) years. Approximately 75% of adults in the ASD group and just over half of those in the ID-only group were male. In the ASD group, 31, 34 and 36% of subjects were noted to have mild, moderate or severe ID, respectively, whereas in the ID-only group, 47%, 35% and 18% of adults had mild, moderate or severe ID, respectively. Most adults in the ASD group (75%) and ID-only group (76%) were white, non-Hispanic.

**Table 4:** Demographic variables among the ASD group and ID-only group in the 2010 to 2011 NCI sample of adults aged 30-59 years

**Demographic variables**	**ASD group**^**a**^	**ID-only group**^**b**^
	**(n = 298), number (%)**	**(n = 3,963), number (%)**
**Age**		
30 to 39 years	148 (50)	1,291 (33)
40 to 49 years	100 (34)	1,473 (37)
50 to 59 years	50 (17)	1,199 (30)
Missing data	0	0
**Gender**		
Female	74 (25)	1,825 (46)
Male	224 (75)	2,137 (54)
Missing data	0	1
**Race/ethnicity**		
White, non-hispanic	224 (75)	3,009 (77)
Black, non-hispanic	51 (17)	738 (19)
Hispanic	14 (5)	128 (3)
Asian	4 (1)	20 (1)
American Indian	1 (0)	32 (1)
Other	3 (1)	17 (0)
Missing data	1	19
**Marital Status**		
Single, never married	296 (99)	3,711 (94)
Single, married in the past	2 (1)	131 (3)
Married	0 (0)	97 (2)
Missing data	0	24
**Level of ID**		
Mild	76 (31)	1,692 (47)
Moderate	85 (34)	1,266 (35)
Severe	87 (35)	637 (18)
Missing data	50	368
**Primary language**		
English	282 (96)	3,880 (98)
Other	12 (4)	67 (2)
Missing data	4	16
**Primary means of expression**		
Spoken	161 (55)	3,387 (86)
Gesture/body language	107 (36)	443 (11)
Sign language/finger spelling	18 (6)	62 (2)
Communication aid/device	3 (1)	21 (1)
Other	6 (2)	22 (1)
Missing data	3	28

^a^ASD group: diagnosis of intellectual disability (ID) and autism spectrum disorder (ASD); ^b^ID-only group: diagnosis of ID but no diagnosis of ASD. NCI, National Core Indicators.

### Outcomes

As in the analysis of the exploratory sample, interaction terms related to age group, gender or level of ID did not provide significant additions to the model for any of the selected outcomes examined (*P* >0.10) for the replication sample. The frequencies of the outcomes across the ASD group and ID-only group also were very similar in the replication sample to those in the exploratory sample. For more detailed figures, please refer to Table 5. PRs, adjusted for possible confounding by age group, gender and level of ID, as well as 95% CIs, are reported in Table 6.

**Table 5:** Outcomes among ASD group and ID-only group in the 2010 to 2011 NCI sample of adults aged 30-59 years

**Outcomes**	**ASD group**^**a**^	**ID-only group **^**b**^
	**(n = 298), number (%)**	**(n = 3,963), number (%)**
**Medication for behavior problems?**		
Yes	124 (44)	847 (22)
No	160 (56)	2,919 (78)
Missing data	14	197
**Limited or no vision-legally blind**		
Yes	13 (4)	208 (5)
No	285 (96)	3,755 (95)
Missing data	0	0
**Severe or profound hearing loss**		
Yes	13 (4)	192 (5)
No	285 (96)	3,771 (95)
Missing data	0	0
**Physical disability**		
Yes	4 (1)	319 (8)
No	279 (99)	3,594 (92)
Missing data	15	50
**Seizure disorder or neurological problem**		
Yes	52 (17)	838 (21)
No	246 (83)	3,125 (79)
Missing data	0	0
**Alzheimer's disease or other dementia**		
Yes	1 (0)	51 (1)
No	297 (100)	3,912 (99)
Missing data	0	0
**Does this person need support to manage self-injury behavior?**		
Extensive	34 (12)	145 (4)
Some	82 (28)	544 (14)
None	173 (60)	3,182 (82)
Missing data	9	92
**Does this person need support to manage disruptive behavior?**		
Extensive	45 (15)	294 (8)
Some	132 (45)	1,077 (28)
None	115 (40)	2,499 (65)
Missing data	6	93
**Does this person need support to manage destructive behavior?**		
Extensive	40 (14)	209 (5)
Some	82 (28)	643 (17)
None	167 (58)	3,013 (78)
Missing data	9	98

^a^ASD group: diagnosis of intellectual disability (ID) and autism spectrum disorder (ASD); ^b^ ID-only group: diagnosis of ID but no diagnosis of ASD.

**Table 6:** Prevalence ratios (ASD group versus ID-only group) for the 2010 to 2011 NCI sample of adults aged 30-59 years

**Outcomes**	**Prevalence ratio (95% CI)**
Medication for behavior problems?	1.15 (1.09, 1.20)
Limited or no vision-legally blind	0.98 (0.96, 1.00)
Severe or profound hearing loss	1.00 (0.98, 1.03)
Physical disability	0.93 (0.92, 0.95)
Seizure disorder or neurological problem	0.95 (0.91, 0.99)
Alzheimer's disease or other dementia	0.99 (0.99, 1.00)
Does this person need support to manage self-injury behavior?	1.89 (1.58, 2.26)
Does this person need support to manage disruptive behavior?	1.62 (1.44, 1.81)
Does this person need support to manage destructive behavior?	1.61 (1.36, 1.90)

Prevalence ratios were adjusted for age group (30 to 39, 40 to 49, 50 to 59 years), gender and level of intellectual disability (ID). ASD group: diagnosis of ID and autism spectrum disorder (ASD); ID-only group: diagnosis of ID but no diagnosis of ASD. NCI, National Core Indicators.

Similar to the exploratory sample, more severe level of ID was significantly associated with disruptive behavior, destructive behavior, limited or no vision and physical disability within the ID-only group but not the ASD group for the replication sample. However, while increasing severity of ID was significantly associated with medication for behavior problems, self-injury behavior and seizure disorder or neurological problem within both diagnostic groups for the exploratory sample, these associations were not significant in the ASD group for the replication sample.

## Discussion

This large-scale, systematic study was designed to examine clinical problems specific to older adults with ASD and ID. Adults with both ASD and ID constitute those individuals with ASD likely to have the greatest degree of behavioral and associated medical problems [19], and therefore are likely to provide insights into those affected adults requiring the greatest levels of care. Context with respect to age-related changes was also provided by contrasting the 50- to 59-year-old age group against subjects aged 30 to 39 and 40 to 49 years. The large size of the overall sample and the ability to examine the same variables using samples from two consecutive years increased confidence in the validity of the presented findings.

No age-associated differences were detected in any of the selected variables between the ASD group and ID-only group across the 10-year age intervals. Similarly, of all the outcomes, age was associated only with hearing loss. Given that the NCI data did not include a sufficient number of subjects aged 60 years and over, the lack of differences in outcomes analyzed by age group may have stemmed from not being able to examine differences in conditions that usually present themselves after age 59 years, when many chronic diseases have their greatest impact. Thus, it simply may be the case that the individuals analyzed in this study were at too premature of a stage in life for the degenerative complications of aging to have occurred.

The most striking result of this study was the high prevalence of severe behavioral problems in subjects with ASD and ID relative to those with ID only. For example, the use of medications for behavioral problems was noted in over 40% of the ASD group in both the exploratory and replication samples. The prevalence of support needed for severe behavioral problems in the ASD group ranged from 37% (for destructive behavior in the exploratory sample) to 60% (for disruptive behavior in the replication sample). In general, severe behavioral disturbances were reported approximately twice as much in the ASD group as in the ID-only group. Although no age-associated changes were revealed, the frequency of problem behaviors requiring intervention in the ASD group was sufficient to raise concern.

The primary conclusion reached in this study, namely, that severe behavioral problems are the consequences of autism, differs from the recent findings of Totsika *et al*. [20]. In that study, older adults with ASD and ID reported a significantly more severe level of behavioral problems than a comparison group of adults with ID only. However, this difference was no longer present after matching for adaptive behavior, leading the authors to conclude that impairment in adaptive skills in older adults with ASD and ID, somehow viewed as independent of the defining features of ASD, was responsible for the increased severity of behavioral problems reported in adults with ASD and ID relative to those with ID only. We view the lack of a significant difference in the severity of behavior problems between the groups as a result of over-matching, as adaptive behavior deficits are likely to be integrally related to the defining features of ASD and not a component that can be meaningfully separated from the condition itself. Similar results appear in a separate study by Melville *et al*. [21], which compared 77 subjects (aged 16 to 84 years) with ASD and ID to 154 individuals with ID only. Although a significant increase in the point prevalence of problem behaviors among those with ASD and ID relative to those with ID only was initially noted, no significant difference in this outcome was reported after a 1:2 matching procedure (due to the relatively small size of the group of individuals with ASD and ID) between the groups on age, gender, intellectual ability and co-morbid Down syndrome. Once again, the vanishing of this significant difference may be attributed to problems in the matching methods. Consistent with the reported findings of the present study, a more recent study comparing 124 adults (aged 18 to 65 years) with ASD and ID to 562 adults with ID only [22], reported an almost four-fold increase in the presence of challenging behavior among those with ASD and ID.

Differences in the methods and ascertainment schemes used in these studies make them difficult to compare. Yet, whereas previous studies have combined subjects across wider age ranges, the present study utilized a considerably larger sample and focused on a more narrowly defined age range (that is, 30 to 59 years). Such inconsistency across the literature ultimately points to the need for prospective studies of the population of uniformly diagnosed, older adults with ASD after systematic ascertainment from sources unbiased with respect to the outcomes being studied.

The high proportions of adults in both diagnostic groups who had behavioral problems noted in this study are consistent with the reports of associated (non-diagnostic) behaviors and co-morbid psychiatric conditions in children, adolescents and young adults with ASD and/or ID [23-29]. One possible interpretation for the increased frequencies of behavioral problems reported in the ASD group relative to the ID-only group is that having problems in behavior may be a direct expression of the biological substrate of autism. Indication of such a possibility is provided through the observation of significant associations between level of ID and disruptive and destructive behaviors within the ID-only group but not the ASD group in both the exploratory and replication samples. Whereas intellectual deficits seem to account for the presentation of behavior problems in the ID-only group, the lack of a significant relationship between level of ID and these types of behaviors in the ASD group suggests that some inherent feature of the actual ASD condition may be responsible for the alarmingly high presentations of behavior problems in adults aged 30 to 59 years with ASD and ID.

An alternative or additional explanation is that the relationship between ASD and associated behavioral problems is indirectly mediated by autistic symptoms or abilities. For instance, social isolation/social deficits may increase the risk for psychiatric or behavioral problems over time. Additionally, caregivers of adults with ASD may not be sufficiently skilled in the care of older individuals with ASD, indirectly contributing to increased rates of behavioral problems. Longitudinal trajectories and the complex interplay of direct and indirect effects on associated behavioral outcomes in autism are areas that have received little attention in the research literature. Investigation into potential mechanisms underlying the high prevalence of behavioral problems in this study is warranted on the basis of the present results.

Given the rapidly expanding population of older adults in western societies, these data raise major public-health concerns about our ability to care for the aging population of adults with ASD and ID. The findings of this study suggest a critical need for research into the assessment and treatment of severe behavioral problems associated with ASD appearing around midlife, along with efforts for training of the workforce in appropriate treatment of this expanding population. These findings further suggest that there will be substantial demands on systems of care required to manage the problems faced by these individuals, their families and communities as they enter residential and nursing care facilities for older adults. The development of health care policies that address family support, institutional care and financing of care for older adults with ASD is, therefore, of critical importance in planning for the care of the aging population of individuals with ASD.

Two outcomes, namely, physical disability and seizure disorder or neurological problem, were slightly less prevalent in the ASD group relative to the ID-only group. No differences were reported in the prevalence estimates for vision or hearing deficits between the groups either. As expected on the basis of findings in the general population, a higher prevalence of hearing impairment was associated with increasing age in both groups, lending support to the validity of this rating. Similarly, no relative differences were observed for the prevalence of Alzheimer’s disease or other dementia. However, early-onset Alzheimer’s disease (before age 60 years) represents less than 5% of all those who are eventually diagnosed with Alzheimer’s disease [30], suggesting that the individuals analyzed in this study may have been too young to adequately examine this relationship.

### Strengths and limitations

Several significant limitations of this study should be noted. The NCI database, spread across many states in the US, does not include unique subject identifiers, making it impossible to examine overlap in annual samples or across states. From discussions with NCI administrators and trainers, although there is an attempt to avoid such redundancy in the sampling for some states, the existence of this problem ultimately cannot be ignored. Another limitation concerns the quality of several of the questions that appear in the NCI data, such as those pertinent to medications that may be associated with particular psychiatric conditions. Standardization across states and raters was not as rigorous as typically seen in studies where data collection was controlled by the investigators, and so these measures were excluded from analyses.

Furthermore, it is important to note that ASD is likely to be under-diagnosed in older populations [31,32], which may have resulted in the inclusion of adults with ASD in the ID-only groups of this study. Overall, the validity of the diagnostic classification, as provided in this administrative dataset, is another limitation of this study. However, the availability of such a large and geographically wide-ranging sampling frame, even given the above noted limitation, strengthened the validity and generalizability of the findings reported here.

### Summary and future directions

Markedl**y** high prevalence estimates of severe behavioral problems in adults with ASD and ID between 30 and 59 years of age are reported in this study. These results have great relevance to policy makers. There is currently no scientific knowledge about mechanisms underlying the behavioral problems noted in this age group and limited knowledge regarding treatment. As seen in the emergence of the specialty fields of geriatric medicine and geriatric psychiatry, the need for the advancement of specific expertise in the care of older individuals with ASD is underscored by the especially high proportions of significant clinical symptoms reported in the ASD group. As knowledge emerges about appropriate strategies for assessment and treatment of the aging population of individuals with ASD, expanded training efforts to build treatment capacity in the community will be essential. Research to identify the most effective intervention and prevention strategies will be required. New approaches to case finding and diagnosis of this age group (where a lack of childhood informants complicates our current diagnostic schemes) will contribute importantly to our ability to provide effective prevention and intervention.

## Conclusions

In summary, a multitude of factors, including the roles for changing social networks and available community support, changes in underlying neurobiology with age and the cumulative effects of life-long symptoms, may all affect later-age outcomes of ASD. Given the likely enormity of the problem and resultant associated costs of managing health concerns in this population of aging adults with ASD, these data highlight the urgency for further research in this area.

## Abbreviations

ASD: Autism spectrum disorder; NCI: National core indicators; ID: Intellectual disability; HSRI: Human Services Research Institute; PR: Prevalence ratio.

## Competing interests

The authors declare that they have no competing interests.

## Authors’ contributions

DK analyzed and interpreted the data and was a major contributor in writing the manuscript. JP conceptualized the project, provided financial support for some of the work, assisted with interpretation of the data and was a contributor to writing the manuscript. MP and LP assisted in the overall ascertainment of the data and interpretation of the variables, through their contact with the instrument developers, and contributed to writing the manuscript. All authors read and approved the final manuscript.
